# Recent Insights into the Control of Human Papillomavirus (HPV) Genome Stability, Loss, and Degradation

**DOI:** 10.3390/jcm4020204

**Published:** 2015-01-27

**Authors:** Chris Fisher

**Affiliations:** NanoVir, 4717 Campus, Kalamazoo, MI 49008, USA; E-Mail: chris.fisher@nanovirpharm.com; Tel.: +1-269-372-3261

**Keywords:** HPV, antiviral, polyamide, antiviral sensitizer, MRE11, viral persistence, TDP1, TDP2, RTEL1, RUVBL2

## Abstract

Most human papillomavirus (HPV) antiviral strategies have focused upon inhibiting viral DNA replication, but it is increasingly apparent that viral DNA levels can be chemically controlled by approaches that promote its instability. HPVs and other DNA viruses have a tenuous relationship with their hosts. They must replicate and hide from the DNA damage response (DDR) and innate immune systems, which serve to protect cells from foreign or “non-self” DNA, and yet they draft these same systems to support their life cycles. DNA binding antiviral agents promoting massive viral DNA instability and elimination are reviewed. Mechanistic studies of these agents have identified genetic antiviral enhancers and repressors, antiviral sensitizers, and host cell elements that protect and stabilize HPV genomes. Viral DNA degradation appears to be an important means of controlling HPV DNA levels in some cases, but the underlying mechanisms remain poorly understood. These findings may prove useful not only for understanding viral DNA persistence but only in devising future antiviral strategies.

## 1. Introduction

The maintenance of stable human papillomavirus (HPV) genomes in cells is the basis for viral DNA persistence, which is the primary risk factor in cervical carcinogenesis [1,2,3]. HPV DNA integration into the host cell genome is also a likely important carcinogenic event. Nearly all cervical cancers show HPV integration in a single chromosomal domain highlighting both the clonal nature of the disease and the growth advantage imparted to cells having integrated high-risk genomes [3]. Therapies that eliminate viral DNA from cells have the potential to curb carcinogenic progression, but the small HPV genome encodes few obvious targets for antiviral intervention. It is therefore important to explore alternative antiviral approaches for HPV.

Viruses have a unique relationship with the cell. They represent a threat to genome integrity that must be overcome, but have evolved the means to circumvent host defenses and to even co-opt host defense elements for their own lifecycle. DNA damage repair (DDR) pathways are at the front line of antiviral defense, yet viruses including HPV have evolved clever means to circumvent and co-opt them. However, recent work with HPV antiviral compounds has made clear that HPV episomes can be massively destabilized and DDR pathways mobilized to facilitate rapid viral DNA degradation and elimination from cells [4,5,6].

DNA degradation is common and fundamental to a number of cell processes including apoptosis and development [7]. A large portion of the human genome is dedicated to encoding DDR pathways that edit and metabolize DNA in order to minimize mutations and other insults to genome integrity. The genome is constantly scanned for mistakes, including DNA lesions caused by chemicals or radiation or the routine business of DNA replication, so that mutations can be minimalized to ensure the health of the cell and its progeny [8]. Viral DNA is susceptible to recognition and removal from cells by degradation. For example, products of HIV reverse transcription are degraded by nucleases such as TREX1 and SLX4 allowing escape from innate immune surveillance [9,10]. Hepatitis B virus (HBV) cccDNA, an episomal replicative intermediate particularly resistant to antiviral drugs, is susceptible to degradation following treatment with interferon or activation of the lymphotoxin β receptor [11].

Previous reviews have covered the current antiviral approaches to HPV therapy that concentrate on targeting, primarily, viral DNA replication [12,13]. These will not be reiterated here. Rather, this review will summarize examples illustrating how HPV DNA may be destabilized leading to large losses of episomes from cells, often accompanied by the apparent degradation of viral DNA and sparing of host cell DNA. What is known of underlying mechanisms will also be considered. These approaches offer a contextual framework for envisioning alternative approaches to eliminating viral DNA episomes from cells that are applicable to HPV and probably other DNA viruses as well.

### 1.1. The Need for HPV Antiviral Therapy

In addition to a high morbidity associated with benign genital warts, an estimated 5% of cancers worldwide are attributable to HPV [14]. HPV is the primary etiological agent in cervical cancers [15], while cancers of the oro-pharyngeal and uro-ano-genital tracks attributable to HPV are also significant and on the rise [16]. HPV vaccines represent an important public health advancement that have the potential to significantly decrease the HPV disease burden worldwide [17]. However, numerous challenges are impeding the worldwide implementation of the vaccines. Factors hindering vaccine coverage include poor delivery to economically disadvantaged populations, the low cost-effectiveness of male vaccination, and the relative low coverage rates achieved in physician-driven, decentralized (non-school-based) settings [17]. Consequently, HPV will be a significant contributor to human morbidity and mortality for the foreseeable future and antiviral therapy for HPV should be aggressively developed. Experience with hepatitis B virus (HBV) provides an important object lesson: over 30 years ago an excellent HBV vaccine was first introduced resulting in a decrease in urgency for development of HBV antiviral therapies. HBV antiviral therapy development lagged and this year nearly one million people will die due to HBV-related disease.

### 1.2. HPV Infection

Human papillomaviruses (HPVs) are highly trophic viruses that infect stratified squamous epithelia including the cutaneous epidermis and the mucosa of the oral-pharyngeal and uro-ano-genital tracks. A subset of the mucosal-infecting HPVs is designated “high-risk” having been implicated as the etiological agents in cervical and other cancers. Over 240 fully sequenced animal papillomaviruses distributed throughout 37 genera, have been identified to date. Of these, ~140 HPVs distributed by sequence homology throughout five of the genera (α, β, γ, μ and ν), are currently recognized [18,19]. The α papillomaviruses are the most studied by far due to their importance in mucosal infection and human disease and because most HPV-maintaining cell lines carry the high-risk α HPV genomes.

**Figure 1:**
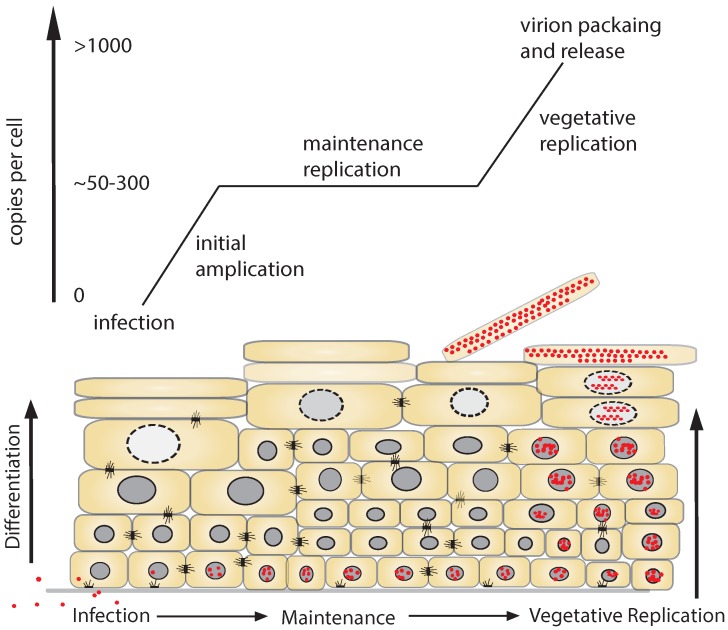
Phases of human papillomavirus (HPV) DNA replication. Following infection, an initial amplification event boosts HPV DNA copy number to 50–300 per cell in the basal, or proliferative, epithelial compartment. Copy number is maintained until the vegetative phase, which occurs during a productive HPV infection. The vegetative (productive) phase is characterized by a second, differentiation-dependent HPV DNA amplification that produces DNA for packaging in virions and sloughing within terminally differentiated cells from the epithelial surface.

The HPV life cycle lacks a lytic phase and has an obligate dependence upon terminal keratinocyte differentiation to produce virions and complete the infectious cycle (Figure 1). Infection is believed to occur through a microabrasion that exposes the proliferative, or basal, cell layer to an HPV virion. Virus attachment and entry occurs most likely via a heparin sulfate proteoglycan receptor [20], which is internalized by an endocytic mechanism that may require clathrin [21,22] or may be a clathrin-independent pathway similar to, but distinct from, micropinocytosis [23], depending upon the cell type studied. Upon cellular entry the virus passes through acidic compartments including late endosomes and lysosomes where the viral DNA is uncoated. The viral capsid protein L2 primarily mediates passage into the nucleus of the viral DNA via a series of cytoplasmic interacting partners [24,25].

### 1.3. Replicative Phases of the HPV Lifecycle

The three replicative modes of the HPV life cycle, establishment, maintenance, and productive replication, have been well characterized *in vitro* (Figure 1) [26]. HPV DNA establishes itself following infection as a chromatinized, replicating, supercoiled, circular DNA, or episome, within the nucleus. Once viral DNA enters the nucleus it is amplified to approximately 50 to 300 copies per cell during the establishment phase in the basal cell layer (Figure 1). These numbers are generally based upon measurements of viral DNA maintained in cultured cells or in infected epithelia [4,27,28,29]. Transient replication of transfected viral DNA has served as a surrogate for understanding viral DNA replication and amplification during the establishment phase, and has allowed for the identification of proteins important for these processes. The viral proteins E1 and E2 (see below) are important for replication and the establishment of viral genomes in this early phase [30,31]. The detection of E1 and E2 transcripts in the earliest stages post-infection with HPV31 virions *in vitro* is consistent with a role in establishment [32]. The E8^E2 gene product also appears to be important in the establishment phase where it plays a suppressive role that limits viral genome amplification and may be important for establishment of some HPV genotypes [33,34,35].

Once established, HPV DNA is maintained long-term within the proliferative basal cell layer at a relatively constant copy number. This maintenance phase of replication is the target of most antiviral strategies since persistent infection by high risk HPV is the primary risk factor for cervical carcinogenesis (Figure 1) [3]. The long-term, stable maintenance of HPV genome copy number in cultured keratinocytes is generally regarded as a model of the maintenance phase of the HPV life cycle. Established viral genomes are kept at a fairly constant number and are replicated during S-phase by utilizing the host cell replication machinery. Replication during the maintenance phase occurs by either an ordered (each episome replicated once per cell cycle) or random (some episomes replicate several times, some don’t) mechanism depending upon the nuclear milieu and E1 levels of the host cell [26,36].

The ability to establish long-term cell cultures that maintain transfected HPV genomes as episomes was an important advancement that has served as a fundamental means to study the maintenance phase of the life cycle [37,38]. Studies using this approach have clearly shown that the viral proteins E1, E2, E6, and E7 are essential for the establishment and subsequent maintenance of viral DNA episomes in host cells [2,37,39,40,41].

The 3′ to 5′ helicase function of the highly conserved HPV protein E1 is key for HPV genome replication and is thought to be central to HPV maintenance [42]. E1 is a classic initiator protein with four conserved domains including an *N*-terminal regulatory region, a DNA binding domain, and oligomerization and ATPase domains. E1 function is dependent upon formation of a hexameric helicase at the origin of replication (*ori*) in cooperation with E2 [42]. Its function is absolutely contingent upon cooperation with the host cell replication machinery with which it interacts closely to promote bidirectional HPV genome replication. The central importance of E1 may be appreciated by the high degree of post-translational regulation controlling its nuclear import and assembly at the origin of replication including CDK2 [43] and MAPK phosphorylation [44], caspase cleavage [45], and interaction with numerous other cell elements [42]. However, debate still exists as to whether E1 is required for episome maintenance replication once the initial establishment phase is completed [46,47].

E2 is a multifunctional protein with crucial roles in viral DNA replication and regulation of transcription that is important for the maintenance of episomes. E2 is likely important for determining stable HPV copy numbers in cells because it has been shown to positively and negatively regulate the early promoter that controls its own expression as well as E6, E7, and E1 [2,48]. A truncated version of E2 (known as E8^E2C) inhibits early gene expression and suppresses HPV replication and copy number lending further support to an important E2 role in controlling stable copy number [35,49]. E2 also has a unique role in fastening HPV genomes to mitotic chromosomes to ensure proper partitioning of viral DNA during cell division [50,51]. Its trans-repressor function for the early promoter regulating E6 and E7 expression is important because loss of E2 expression following HPV DNA integration is understood to contribute to carcinogenesis via up-regulation of oncogene expression [52,53,54]. Importantly, reintroduction of exogenous E2 into cervical cancer cells carrying integrated HPV genomes (without functional E2 genes) restores transcriptional control of the E6 and E7 oncogenes ultimately resulting in apoptosis or senescence [55,56,57]. These observations effectively demonstrate that apoptotic and senescence pathways remain intact, but suppressed by viral oncogenes, in invasive cervical cancer.

The E7 oncoprotein promotes cell cycle progression and upholds replication competence during maintenance in basal cells by targeting the pRb tumor-suppressor protein for destruction and through interactions with a host of other cell elements that include activation of CDK2 [58,59,60]. Similarly, the oncoprotein E6 is a multipurpose protein that, in addition to its key role in inactivation of p53, also blocks apoptosis and controls various cellular processes such as immune evasion while advancing an extended life span through induction of telomere maintenance via hTERT [61]. E6 and E7 together promote large changes in the epithelial transcriptome to promote an environment in which cell cycle progression is controlled to enhance viral DNA maintenance and productive replication, and to protect against senescence through an extension of the host cell lifespan [62].

A second amplification phase occurs during a productive infection (*i.e.*, in a wart) in which infectious virions are produced (Figure 1). As cells differentiate and move from the basal layer through the suprabasal layers of the epithelium, a dramatic increase in transcripts for E1, E2, E1^E4, E5, and E8 occurs [63,64,65] resulting in significant amplification of viral episomes [29,66]. This productive (or vegetative) amplification phase results in at least a 2-log amplification of DNA per cell in tissues [28,29] although *in vitro* the amplification is more modest [27,66,67,68] perhaps due to the inability of *in vitro* systems to support a complete program of cell differentiation. Viral DNA amplification following keratinocyte differentiation induced *in vitro* either by cell suspension in methylcellulose, withdrawal of growth factors, or in organotypic cultures, has provided a useful means to measure this replicative phase. The viral oncogenes E6 and E7 are important for DNA amplification during this stage because they contribute to the creation of a milieu supporting cell cycle progression and DNA synthesis in the differentiated, post-mitotic cell [62,69,70,71].

### 1.4. DNA Damage Response (DDR) and DNA Viruses

DNA damage response (DDR) pathways are barriers that must be overcome by DNA viruses, a feat that is accomplished in a number of elegant and surprising ways. A comprehensive summary of DDR and DNA viruses is beyond the scope of this review but the area has been well covered by excellent reviews [72,73,74]. The ataxia-telangiectasia mutated (ATM) and ATM and Rad3-related (ATR) serine/threonine protein kinases sense DNA damage [8]. ATM senses and organizes the cellular response to dsDNA break repair, while ATR organizes the DDR to a wider variety of DNA insults such as exposure of ssDNA and stalled replication forks. The Chk2 and Chk1 effector kinases act downstream of ATM and ATR, respectively, to help coordinate and implement the cellular response [75].

Elements of both the ATM and ATR pathways are activated in HPV positive cells, and a role for ATM activation has been implicated in productive HPV DNA replication [69,76,77,78,79]. One way that this is accomplished is through regulation of caspases, which cleave HPV E1 [45,76]. E1, modulated by E2, notably activates ATM [77,78], which can also be activated by HPV E7 via STAT-5 [76,80]. A number of homologous recombination pathway members have been localized to HPV replication centers suggesting that they, in addition to ATM, are important for HPV episome amplification via recombination-dependent replication [76,77,79,81]. For recent reviews of the regulation of differentiation-dependent episome amplification see: [29,71].

## 2. HPV Episome Stability and Degradation

As briefly discussed above, studies of the HPV maintenance phase have mostly focused on the ability of genomic DNA to establish episomes in long-term cultures. Many studies have established which virus-encoded proteins and interacting partners are required to support long-term HPV genome maintenance. However, several observations have been made that indicate HPV episomes are subject to another level of control that is less well understood and in need of study: that is, the active loss or clearance of HPV episomes from cells. This area is of high importance because elimination of viral episomes from cells is the goal of most antiviral approaches.

### 2.1. Interferon

Interferon-α, -β, and -γ therapies for HPV infections have been widely employed although unwanted side effects limit their clinical potential [82]. Interferon treatment was first noted to cause the elimination of bovine papillomavirus-1 (BPV-1) from mouse C-127 cells resulting in the recovery of “cured” cells [83]. Similarly, treatment with interferon-β causes the rapid loss of HPV16 episomes (~80% in eight days) from W12 cells by poorly understood mechanisms attributed to “innate immunity” [84,85]. This short-term loss of episomes results in the outgrowth of cells containing only integrated copies of HPV16, and thus provides a practical *in vitro* model for HPV-dependent carcinogenesis in which loss of episomes expressing the E2 transrepressor permits viral oncogene up regulation and emergence of a transformed phenotype [53,84,86]. Another study conducted in HPV31 maintaining cells largely agrees that interferon-β triggers the loss of episomes although the time course was longer and measured over passages rather than days [85]. Such studies are difficult to conduct due to the fact that IFN triggers cell death in cells maintaining episomes. Nevertheless, the reports demonstrate that episomal elimination occurs in response to interferon treatment and the time-course of the events strongly suggests HPV DNA elimination is an active process. It is of high interest, therefore, to identify those pathways that interferons engage to trigger episome loss since they may possibly be implemented in therapeutic strategies.

As protection against this cellular response, HPV encodes proteins that can disrupt interferon signaling in a number of ways including interfering with the Janus kinase-signal transducer and activator of transcription (JAK/STAT) pathways, a major mediator of interferon signal transduction [87]. E6 has been shown to interfere with Tyk2 kinase [88] and interferon-regulatory factor 3 (IRF3) [89], while both E6 and E7 transcriptionally repress STAT-1 [90]. Restoration of STAT-1 levels in cells maintaining HPV31 episomes resulted, like interferon, in the loss of episomes and selection for cells with integrated HPV DNA [90], however the mechanism underlying episome loss was not explored. Supporting evidence of a role for STAT-1 in cell-mediated episome loss comes from studies pertaining to the apolipoprotein B mRNA editing enzyme, catalytic polypeptide-like 3 (APOBEC3) cytidine deaminase family of proteins.

The APOBEC3 proteins have a wide variety of functions that include generation of diversity in the immune system in cooperation with various DDR pathways and the clearance of foreign DNA from cells [91,92]. APOBEC3 appears to be a plausible candidate for mediating HPV episome loss considering that its expression is transcriptionally regulated by STAT-1 [93]. The APOBEC3 family of seven proteins is responsible for restricting a wide variety of foreign or invasive genetic elements including endogenous retroelements and multiple RNA and DNA viruses, and has been shown to mediate the clearance of foreign DNA from cells [92,94]. Interestingly, the long control region (LCR) of HPV16 is hypermutated by APOBEC3 in cervical precancerous lesions as is the HPV1a LCR when transfected together with APOBEC3 expression vectors in 293 cells [95]. APOBEC3A and APOBEC3B are up-regulated in HPV-positive cells and cervical tissue, and APOBEC3A acts to restrict HPV infection [96]. Treatment of W12 cells with interferon-β results in up-regulation of *ABOBEC3* gene expression and hypermutation of the HPV16 E2 gene in a manner dependent upon inhibition of uracil DNA glycosylase (which repairs the APOBEC3-mediated mutation) [97]. Since APOBEC3s cause HIV DNA degradation through a uracil DNA glycosylase-dependent base excision repair (BER) pathway, the effects of uracil DNA glycosylase inhibition on interferon-β-mediated loss of HPV episomes were examined, but no effect on episome loss was found over the course of 72 h [97]. Thus, while APOBEC3 enzymes are unregulated in HPV-infected cells and tissues where they may act as restriction factors, and APOBEC3 editing of HPV episomes appears to be triggered by interferon-β, the modifications do not appear to play a critical role in controlling HPV episomal levels or stability. These observations contrast with the recent demonstration that interferon-α mediated hepatitis B virus cccDNA loss and degradation in the absence of hepatocyte toxicity is dependent upon ABOBEC3A and ABOBEC3B activity [11,98].

IFN-induced protein with tetratricopeptide repeats 1 (IFIT1) is another novel mediator of HPV episome loss that is similarly worth considering. IFIT1, also regulated by the JAK/STAT pathway, is specifically down regulated in cells carrying high-risk HPV episomes [99]. IFIT1 (also known as ISG56 or P56) binds the HPV E1 C-terminus through its *N*-terminal tetratricopeptide repeat 2 domain and, in doing so, inhibits E1 helicase activity, E2 binding, and sequence-specific HPV *ori* DNA binding [100,101]. These IFIT1 effects play an important role in the interferon response by inhibiting HPV replication. It should also be pointed out that, as a mediator of E1 replicative function, IFIT1 has the potential to also influence HPV episome stability (see Section 3, below) although this has not been reported.

### 2.2. Antiviral Polyamides (AVPs) that Promote Massive HPV Episome Instability and Degradation

**Figure 2:**
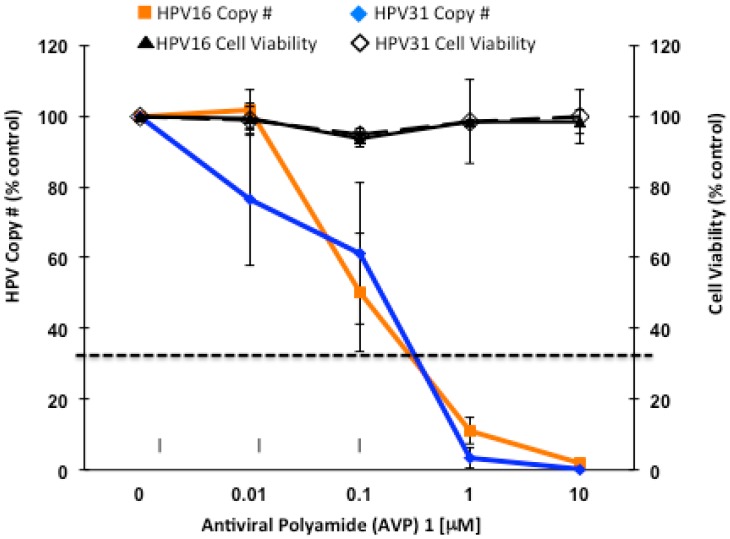
Antiviral polyamide 1 potently induces rapid loss of HPV episomes in the absence of cytotoxicity. The 48 h. dose response curves show loss of episomes for two different viruses (HPV16 and HPV31) while at the same time exhibiting no cell toxicity. Note that AVP1 drives episomes to nearly undetectable levels. The black, dashed line represents theoretical viral DNA levels following treatment with a putative “perfect” viral replication inhibitor after two cell cycles in 48 h.

Distamycin A and other naturally-occurring, minor-groove DNA binding agents have long been considered as anti-infective agents including their potential as antivirals against DNA viruses [102,103]. However problems such as toxicity and lack of specificity have limited their clinical usefulness. Higher order, synthetic homologs of distamycin A (*N*-methylpyrrole-imidazole polyamides) designed to bind to AT-rich regions within the origin of replication of high-risk HPVs, possess the remarkable ability to induce rapid loss of HPV episomes in the absence of cytotoxicity (Figure 2) [4,5,6].

In contrast to distamycin A, which causes episome loss at high concentrations but with closely associated cytotoxicity, antiviral polyamides (AVPs) trigger the noncytotoxic loss of HPV episomes well beyond what would be predicted for inhibition of viral replication over an equivalent time course (Figure 2) [4,5,6]. AVPs have been routinely tested and demonstrated no cytotoxicity up to concentrations of 200 µM, which approach the limits of solubility of the compounds [5]. For this reason, since a 50% cytotoxic concentration (CC_50_) has never been achieved, the selectivity index (SI = IC_50_/CC_50_) for AVPs can only be theoretically estimated. Using such estimates, the SI for AVPs is predicted to be >1000-fold superior to that of distamycin A [5]. For this reason it is apparent that AVPs, which were originally designed to block E1- and E2-mediated HPV replication by targeting their binding sites in the viral origin of replication, are acting by a novel mechanism. It is clear that HPV genomes, therefore, can be specifically targeted for elimination while sparing the host cell genome since elimination of episomes in response to AVPs occurs in the absence of measurable toxicity or significant alterations in cell cycle progression.

The rapid destruction of HPV episomes by AVPs in the absence of cytotoxicity is highly unusual, and therefore investigations of their mechanism of action are illuminating. Cell replication or cell cycle progression is not required for AVP antiviral activity since arrest of cells in S-phase or M-phase has no effect on their activity [4]. It is therefore unlikely that generation of unstable DNA replication intermediates or replication stress is important for AVP effects on episomes. In addition, no changes in the expression of interferon-responsive genes occur in response to AVPs (Terri Edwards and Chris Fisher, unpublished observations), indicating that they trigger viral DNA elimination outside of the framework of cytokine mediators of innate immunity. On the other hand, AVPs induce robust transcriptional changes within DNA damage response (DDR) pathways as demonstrated in a series of PCR array experiments [6].

### 2.3. A Role for DNA Damage Response (DDR) Pathways in HPV Episome Stability, Loss, and Degradation

**Figure 3:**
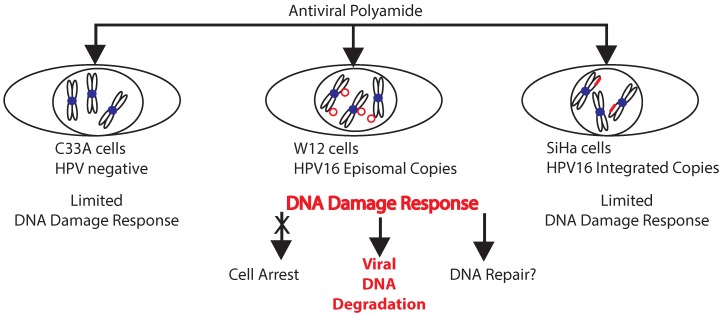
Specificity of antiviral polyamides for cells containing HPV episomal DNA [6]. Treatment of HPV-negative (C33A) and HPV16-integrated (SiHa) cells with AVP causes a limited, nearly identical transcriptional alteration of DDR genes. On the other hand, AVPs elicit large transcriptional changes in DDR pathways in cells maintaining HPV16 episomes. The DNA damage response does not result in cell cycle arrest but does lead to degradation and loss of HPV DNA. Repair of HPV DNA is not evident in Southern blots. However, the enhancement of viral DNA loss and fragmentation caused by inhibition of MRE11 suggests that the MRN complex acts to oppose AVP-mediated destruction of episomes.

The specificity of AVPs for binding episomes and signaling through DDR pathways is shown in studies of AVP effects on cellular transcripts. Only cells carrying HPV episomal DNA exhibit robust activation of DDR pathways in response to AVPs (Figure 3). In contrast, neither HPV negative cells (C33A) nor HPV16 positive cells carrying only integrated copies (SiHa), display a significant DDR response to AVPs (Figure 3) [6]. Moreover, changes in host cell DDR gene expression are not observed when episome-maintaining cells are treated with inactive polyamides (Figure 3). These results help establish that the interaction of AVPs with viral DNA episomes, not host DNA or integrated viral DNA, is important for eliciting a large DDR that may contribute to elimination of viral episomes.

A report found that smaller polyamides of the same family as AVPs cause replication stress resulting in ATR activation, cell cycle arrest, and toxicity [104]. These results appear to have little bearing upon the activity of AVPs, which are not toxic, have equivalent antiviral activity even in non-dividing cells, and are not dependent upon the ATR/CHK1 pathway for their antiviral activity [4]. Further discussion of the ATR/CHK1 pathway as it relates to episome stability is provided below in Section 3.

What might explain the specificity of large AVPs for HPV episomes? Polyamides and other minor groove-binding agents alter DNA structure in a number of ways that include expanding the minor groove, shrinking the major groove, and stiffening DNA [105,106,107]. Such effects predictably alter the cooperative binding of protein complexes to DNA [108,109]. AVPs also exhibit promiscuous DNA binding *in vitro*, compared to shorter polyamides, which may contribute to their antiviral activity [110,111]. Since AVPs are predicted to bind a minimum of 10 base pairs (or one helical turn) of DNA, it is possible that binding within the supercoiled episome results in uneven distribution of turns, exposure of ssDNA, and the appearance of unusual topological structures, or even DNA strand breaks, that would trigger a DDR. The small, circular nature of the HPV genome may exacerbate these effects by limiting their dissipation away from AVP binding sites. The rapid and transient phosphorylation of RAD9 in episome-bearing cells, but not in HPV-negative cells, following administration of AVPs is consistent with this premise [4]. RAD9 is a member of the trimeric RAD9-HUS1-RAD9 (9-1-1) complex, a processivity factor related to PCNA that acts to bring DDR elements to sites of DNA damage [112,113]. RAD9 is therefore a good candidate for coordinating a DDR response to AVP binding that ultimately results in HPV episome destruction.

Notably, AVP selectivity for HPV episome-containing cells mirrors that of interferon-β. Interferon-β exerts profound effects on cell growth and viability in cells that carry HPV31 episomes, but not normal keratinocytes or HPV-negative squamous cancer cells [85]. It is interesting, therefore, to speculate that AVPs and interferon selectivity may be due to the low level DDR that is already present in episome-bearing cells. As mentioned previously, multiple HPV genes are able to activate DDR pathways resulting in HPV-positive cells with heightened DDR activity [4,76,79]. Consequently, HPV-episome bearing cells may be primed to respond to interferon-β by triggering cell death, and to AVP-induced changes in HPV episomal DNA structure by triggering episome destruction.

DDR pathways are complex with many elements playing unique and overlapping roles in response to various insults. The MRE11-RAD50-NBS1 (MRN) complex plays a central role in a variety of processes involving repair of DNA ends in such diverse structures as telomeres, replication forks, and dsDNA breaks [114]. MRN acts as a recruiter and activator of ataxia-telangiectasia mutated (ATM) at DNA ends where the kinase plays a central role in organizing and effecting DNA repair [8]. Among the genes whose expression is most significantly altered by AVPs are MRN complex members MRE11 and NBS1 as well as CtIP, an endonuclease that is required for efficient MRN-mediated homologous recombination and is essential for dsDNA break resection [6,115]. ATM and 9-1-1 complex member RAD9 are also transcriptionally altered by AVPs (RAD9 showed the greatest fold change of all genes examined) as are three Fanconi Anemia pathway genes: *FANCB*, *FANCC*, and *FANCL* [4]. The alteration of *FANC* gene expression in response to AVPs is of interest because members of Fanconi Anemia pathways appear to act as HPV suppressors in laboratory studies [116,117] and mutations within the pathway may be associated with high rates of squamous cell carcinoma [118].

### 2.4. Enhancers and Repressors of AVP-Mediated HPV DNA Instability

A focused, unbiased siRNA screen of 240 *DDR* genes has identified enhancers and repressors of AVP activity [6]. The screen, though focused, was a large study employing four complete replicate experiments, conducted on four separate days, which enabled the identification of effective genes with a high degree of statistical confidence. A total of 16 AVP repressors and four AVP enhancers were identified in the study. Repressors are those genes whose knockdown caused AVPs to more efficiently eliminate HPV DNA, while siRNAs targeting enhancers diminish AVP activity. *MRE11* is among the significant genetic repressors identified. Two 9-1-1 complex members are also important modulators of AVP activity: *RAD1* is a repressor (identified also as the most transcriptionally altered *DDR* gene by AVP; see above) and *RAD9* is an enhancer (found also to be phosphorylated in response to AVPs, see above).

### 2.5. Identification of an AVP Sensitizer

**Figure 4:**
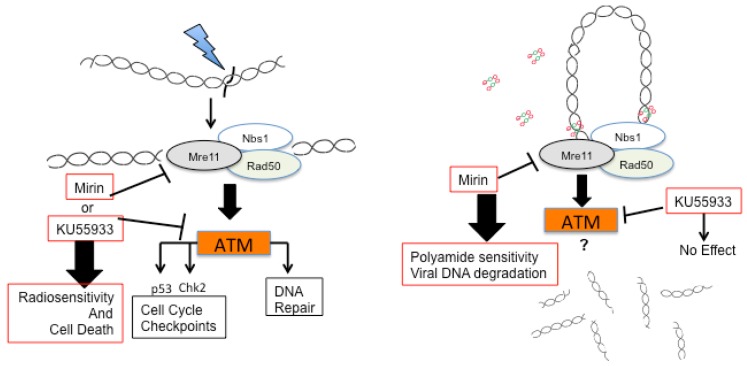
Mirin is an antiviral polyamide (AVP) sensitizer. Inhibition of MRE11 promotes the effectiveness of chemo- and radio-therapy (left) and antiviral polyamides (AVPs) (right). Inhibitors of DNA repair processes increase cell sensitivity toward therapeutic irradiation and chemotherapeutic agents that act by damaging cellular DNA. Inhibition of, for example, MRE11 or ataxia-telangiectasia mutated (ATM) augments cell death in response to DNA damage by preventing cell cycle arrest and DNA repair. In an analogous manner, Mirin (but not ATM inhibitor KU55933) promotes the degradation of HPV episomal DNA in response to AVPs. MRE11 therefore has a role that protects viral episomes from AVPs that does not require signaling through the ATM/CHK2 pathway [6].

Mirin is an inhibitor of MRE11 exonuclease activity that also blocks MRN activation of ATM during dsDNA break repair [119,120]. DNA damage repair inhibitors, including ATM and MRE11 inhibitors, increase the effectiveness of DNA-damaging cancer chemotherapeutic drugs by interfering with DNA repair [121,122,123]. In a surprisingly analogous fashion, Mirin increases the effectiveness of AVPs (Figure 4). Mirin not only enhances the loss of HPV genomes triggered by AVPs, it also lowers the AVP antiviral IC_50_. Importantly, MRE11 inhibition by Mirin results in enhanced degradation of episomes in response to AVPs. This is observed as an increase in the number of HPV DNA strand breaks as measured by end-labeling and capture of DNA followed by HPV-specific Q-PCR [6].

MRE11 and the MRN complex, therefore, protect HPV genomes from destruction prompted by AVPs. MRE11 protection does not require signaling through ATM since inhibition of its kinase activity with the specific kinase inhibitor KU55933 has no effect on AVP activity (Figure 4) [6]. Other ATM-independent roles for MRE11 have been described including stabilization of dsDNA breaks by end-bridging, processing of the ends of dsDNA breaks to facilitate CtIP recruitment, homologous recombinational repair, and microhomology-mediated end joining [114,124,125].

Although MRE11 plays a role in the stability of viral episomes in the presence of AVPs, it is unclear what cues might initially engage it. It is also not known whether the binding of AVPs to episomes directly causes DNA breaks within the supercoiled episome. Another possibility is that DNA repair complexes are recruited to HPV episomes to excise those DNA regions bound to the large AVP adduct and thereby enzymatically introduce DNA breaks.

## 3. ATR and CHK1 Stabilize Episomes during the Maintenance Phase

The DNA polymerase inhibitor aphidicolin has no effect on AVP destabilization of HPV episomes but causes significant loss of episomes by itself [4]. Degradation is the likely reason for loss of viral DNA since it occurs in the absence of DNA synthesis (aphidicolin being a polymerase inhibitor) and is therefore not attributable to selective inhibition of viral DNA replication.

**Figure 5:**
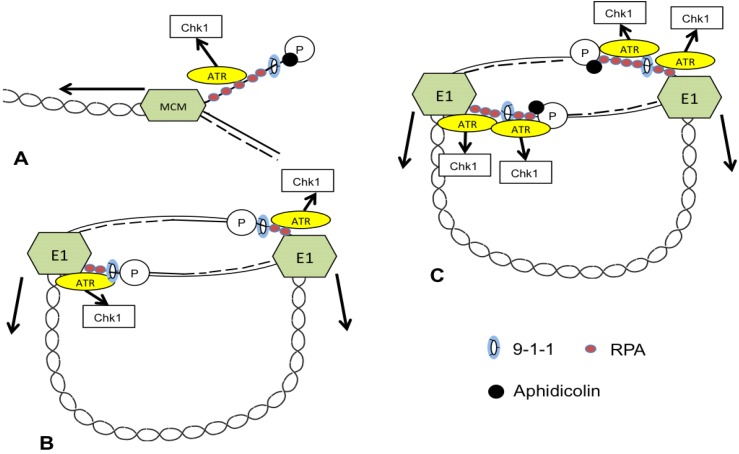
Putative model for instability of HPV episomes in presence of ATR/CHK1 inhibitors or aphidicolin. Simplified models of vertebrate replication fork in presence of aphidicolin (**A**) and HPV replication fork in the absence (**B**) or presence (**C**) of aphidicolin are shown. (**A**) Aphidicolin inhibition of polymerase in vertebrate cells results in continued activity of the MCM helicase, exposure of ssDNA, and activation of the ATR/CHK1 pathway to stabilize the fork; (**B**) E1 activity reportedly activates ATR and ATM, and inhibition of ATR/CHK1 results in loss of episomes suggesting that that ATR normally stabilizes viral replication fork; (**C**) It’s proposed that polymerase inhibition by aphidicolin along with continued E1 helicase activity results in additional exposure of ssDNA, activation of ATR/CHK1 pathway, and accumulation of unstable replication intermediates which are degraded by the cell.

Since aphidicolin causes loss of HPV episomes, it was reasoned that inhibition of ATR and its downstream effector CHK1 might also cause episome loss (Figure 5). The serine/threonine protein kinases ataxia telangiectasia mutated (ATM), ATM and Rad3-related (ATR), and DNA-dependent protein kinase (DNA-PK), and the down-stream effectors CHK2 and CHK1, are important sensors and mediators of DNA damage [8]. ATM responds to dsDNA breaks (DSBs), while the ATR pathway is known to stabilize stalled replication forks. Therefore the question was asked if destabilization of HPV replication forks by inhibition of ATR or CHK1 would also lead to HPV DNA degradation. ATR siRNA knockdown and CHK1 inhibitors caused significant loss of episomes in cells maintaining HPV16, 18, or 31 to a similar degree as aphidicolin indicating that loss of ATR/CHK1 signaling also leads to episome loss in undifferentiated cells, most likely due to degradation.

HPV E1 activates both ATM and ATR resulting in arrest in early S-phase, which is detrimental to cell proliferation, viability, and episome maintenance. For this reason, E1 nuclear accumulation is tightly controlled by post-translational means [77,78,126]. The rapid loss of HPV episomes following inhibition of ATR or CHK1 demonstrates that the pathway plays an important, stabilizing role during normal maintenance presumably because the HPV replication fork maintained by HPV E1 and host replication factors is intrinsically unstable. Aphidicolin further destabilizes the viral replication fork resulting in HPV DNA loss and degradation (Figure 5).

## 4. Additional Mediators of Episome Stability

The large DDR siRNA screen that identified repressors and enhancers of AVP-mediated HPV episome instability/degradation also identified a number of genes, with a high degree of statistical confidence, that play a role in maintaining normal HPV episome levels [6].

Multiple components of the ATM pathway localize to HPV replication centers. While ATM inhibition does not interfere with episome maintenance, it has been shown to be required for productive amplification [4,76,79]. Surprisingly, ATM knockdown does result in a large loss of episomes from undifferentiated keratinocytes indicating that the large ATM protein also plays an important HPV DNA stabilizing role that does not require kinase activity [4,6]. Catalytically inactive ATM causes genomic instability and embryonic lethality in mice demonstrating the importance of additional, poorly understood roles for ATM beyond its kinase signaling activity [127,128].

The tyrosyl-DNA-phosphodiesterases TDP1 and TDP2 are both highly significant effectors of HPV episome stability in an siRNA screen of the DDR pathway: TDP1 knockdown caused a large gain in episome copy number while TDP2 knockdown caused a large loss [6]. TDP1 and TDP2 were originally discovered and named for their ability to process DNA ends by excising the irreversible tyrosyl-DNA bonds formed by topoisomerases I and II, respectively, in the presence of topoisomerase poisons [129,130]. It is now recognized that the workload of these enzymes is much greater. TDP1, in coordination with the base-excision repair pathway, resolves 3′ tyrosines and 3′-phosphoglycolates caused by oxidative damage as well as a variety of other physiological and pharmacological 3′ blocking lesions. TDP2 on the other hand, which coordinates with members of the non-homologous end-joining repair pathway, has specificity for 5′ topoisomerase II complexes. TDP2, also known as TTRAP, has also been shown to be important in signal transduction pathways [130,131]. More recently TDP2 has been implicated in the picornavirus life cycle as the VPg unlinkase that cleaves a protein-RNA covalent linkage generated during viral genomic replication [132], and in the formation of hepatitis B virus cccDNA formation from relaxed circular DNA [133]. The apparently oppositional roles of TDP1 and TDP2 in controlling HPV episome levels suggests these enzymes are potentially valuable subjects of study for understanding HPV episome persistence.

Two helicases involved in homologous recombination, RUVBL2 and RTEL1, were also determined to stabilize HPV episomes: knockdown of each resulted in a greater than 3-fold loss of HPV genomes [6]. RUVBL2 (Reptin, TIP48, or RVB2) is a human homolog of the bacterial *RuvB* gene which appears in many cellular protein complexes including the chromatin remodeling INO80 complex and is implicated in a variety of processes including transcription, DNA repair, cell division, and the exchange of histones [134,135]. RTEL1 is a Fe-S helicase that dismantles DNA secondary structures such as D-loop intermediates and G4-DNA structures arising during DNA replication, repair, and recombination [136]. Both RUVBL2 and RTEL1 are also implicated as important for homologous recombination and in telomere maintenance [137,138].

Interestingly, many statistically significant genes identified in the DDR siRNA screen [6] have been previously implicated in HPV-related biology or disease highlighting the physiological significance of the siRNA screen (Table 1) [6].

**Table 1:** Summary of Mediators of Episome Stability and Previously Identified Roles in HPV Biology.

Gene	Effect on HPV Episomes [6]; Function	Other Known HPV Roles
*MGMT*	Repressor of AVPs; Base excision repair methyltransferase	Proteolytic Target of E6 [139] Promoter methylated in cervical cancer [140]
*TP73*	Repressor of AVPs; Tumor suppressor; Transcription Factor (P53 family)	Promoter methylated in cervical cancer [140]; linked to cervical carcinogenesis [141]
*MLH3*	Repressor of AVPs; knockdown decreases normal HPV episome levels; mutL homolog	Associated with risk of HPV infection and cervical carcinogenesis [142]
*TYMS*	Repressor of AVPs; Thymidylate synthetase	Associated with infection by high-risk HPV [143]
*FAN1*	Repressor of AVPs; FANCD2-associated nuclease; Fanconi anemia pathway	Fanconi anemia pathways implicated in HPV infection [116,144]
*FANCC*	Knockdown decreases normal episome levels; Fanconi anemia pathway	Fanconi anemia pathways implicated in HPV infection [116,144]
*FANCF*	Knockdown increases normal episome levels; Fanconi anemia pathway	Fanconi anemia pathways implicated in HPV infection [116,144]
*MTOR*	Knockdown decreases normal episome levels; kinase, central regulator	MTOR activated by HPV E6 [145]; implicated in HPV infection/entry [146]

## 5. TREX1: A Candidate Mediator of Episome Degradation

Interferon, AVPs, and instability of the HPV replication forks all result in degradation of HPV episomes. It is clear that polyamides trigger damage within episomes in the form of DNA breaks, and that HPV DNA genomes are then rapidly eliminated from cells. Interestingly, Southern blotting does not detect evidence of DNA repair in the form of concatemers or other intermediates that might be detected if repair, and subsequent end joining of viral DNA breaks, were occurring. Instead, viral episomes are rapidly eliminated, suggesting that a decision is made by the cell to destroy, rather than repair, the viral DNA. Little is known of how the viral DNA is ultimately destroyed although it is evidently rapid since observation of degradation intermediates is a rare event. Double-strand DNA breaks are highly toxic and subject to a high degree of regulation. Non-homologous and homologous recombinational repair pathways, as well as telomere protection pathways [147], all conspire to ensure that dsDNA breaks only occur fleetingly in viable cells. For this reason it is not surprising that once a commitment to viral DNA degradation is entered that it is efficiently enforced.

The 5′–3′ exonuclease TREX1 is a candidate “executioner” nuclease for this final step. TREX1, the most abundant exonuclease in mammalian cells [148,149], was found to be transcriptionally up-regulated after addition of AVPs to cells [6]. TREX1 plays a role in the normal metabolism and clearance of self-DNA so that activation of interferon pathways and production of autoantibodies is avoided. Consistent with this role, TREX1 mutations cause the autoimmune disorders Aicardi-Goutieres syndrome (AGS) and chilblain lupus due to accumulation of cytosolic DNA [150,151]. There is also precedence for TREX1 involvement in clearing viral DNA: TREX1 is exploited by HIV to clear cytosolic DNA produced by reverse transcription and thus evade detection and immune surveillance [9,148]. TREX1 is a member of the SET complex along with two other nucleases (endonucleases APE1 and NM23-H1), the chromatin modifying proteins SET and pp32, and HMGB2, a DNA binding protein that recognizes distorted DNA [152]. The SET complex is mobilized during Granzyme A-mediated cell death to act in concert with NM23-H1 to degrade DNA [153]. Whether TREX1 or other nucleases are identified as the agents of HPV viral DNA clearance following destabilization, it will be important to understand their action as they represent potentially important agents of antiviral activity that may be therapeutically important.

## 6. Caveats of Viral DNA Integration

Antiviral strategies to eliminate viral DNA should be given strong consideration since (as discussed above) HPV persistence is the most important risk factor in HPV-related carcinogenesis. However, treatments that eliminate HPV episomal DNA have the potential to cause or exacerbate the effects of HPV integration and therefore need to be developed with that in mind. Disruption of the HPV E2 open reading frame during viral integration, with concomitant loss of the E2 protein and its transrepressor function, results in up-regulation of the HPV E6 and E7 oncogenes and subsequent loss of cell growth control [52,53,154,155,156,157]. Integrated copies of HPV with a disrupted E2 reading frame may exist in a repressed state within populations of cells due to the presence of intact HPV episomes that provide functional E2 protein. In this case, elimination of viral episomes has the potential to result in up-regulation of viral oncogene expression and subsequent genomic instability and carcinogenic progression. This scenario has been confirmed in the W12 cell model system. Treatment of W12 cells with IFN-β causes the rapid loss of episomes, de-repression of the E6 and E7 oncogenes, and the emergence of cells carrying only integrated copies of HPV16 [84]. Thus, IFN-β treatment does not cause integration in W12 cells, but it does enable the rapid expansion of cells with pre-existent integrated viral DNA [84]. AVPs have not been examined in the long term for their ability to enable outgrowth of cells carrying integrated HPV.

Antiviral treatments may also have the ability to promote viral DNA integration, and therefore attention must be given to this potential downside. It’s well established that HPV DNA preferentially integrates into fragile sites [158,159]. It is noteworthy that both fragile sites and HPV episomes exhibit instability under many of the same conditions including aphidicolin treatment and loss of ATR/CHK1 signaling [4,160], and that HPV may replicate in close proximity to fragile sites [161]. Moreover, an increase in the incidence of dsDNA breaks contributes to an increased propensity for integration of HPV, or marker, DNA [162,163,164].

For these reasons it is important to determine if integration occurs following antiviral treatments that destabilize and cause loss of episomes. Southern blots of HPV DNA from cells treated with AVPs do not show evidence of viral DNA integration [5,6]. Likewise, cells treated with both Mirin and AVPs (Terri Edwards and Chris Fisher, unpublished results) do not show evidence of integration in Southern blots of HPV DNA, nor has integration been detected in cells in which episomes have been destabilized by aphidicolin or inhibition of ATR or Chk1 [4]. These results are encouraging although longer-term studies utilizing more sensitive techniques for identifying integrants will need to be conducted.

## 7. Conclusions

The small HPV genome encodes few conventional antiviral targets but alternative approaches to eliminating viral genomes are emerging. Future antiviral strategies will be informed by an understanding of how cells might be prompted to eliminate viral DNA. Interferons have long been employed to treat viral diseases including HPV, while AVPs now allow for the chemical control of viral DNA levels. Both act to eliminate viral DNAs from cells by mechanisms that are beginning to be understood. Genes and pathways have been identified that stabilize HPV genomes against loss and degradation. These elements may have potential as antiviral targets themselves or as sensitizers to enhance antiviral agents as has been shown with AVPs. The identification of genetic AVP enhancers and suppressors, and the discovery of an AVP sensitizer, illustrates how mechanistic knowledge of antiviral agents can be exploited to improve their effectiveness. It is likely that other small DNA viruses will also be vulnerable to these or similar approaches.
